# Iron Inhibits Respiratory Burst of Peritoneal Phagocytes *In Vitro*

**DOI:** 10.5402/2011/605436

**Published:** 2011-12-08

**Authors:** Kamil Gotfryd, Aleksandra Jurek, Piotr Kubit, Andrzej Klein, Bohdan Turyna

**Affiliations:** ^1^Department of General Biochemistry, Faculty of Biochemistry, Biophysics and Biotechnology, Jagiellonian University, Gronostajowa 7, 30-387 Cracow, Poland; ^2^Molecular Neuropharmacology Group, Department of Neuroscience and Pharmacology, Faculty of Health Sciences, University of Copenhagen, Blegdamsvej 3, Building 18.6, 2200 Copenhagen N, Denmark; ^3^Department of Nephrology and Dialysis, Rydygier Hospital, Złota Jesień 1, 31-826 Cracow, Poland

## Abstract

*Objective*. This study examines the effects of iron ions Fe^3+^ on the respiratory burst of phagocytes isolated from peritoneal effluents of continuous ambulatory peritoneal dialysis (CAPD) patients, as an *in vitro* model of iron overload in end-stage renal disease (ESRD). *Material and Methods*. Respiratory burst of peritoneal phagocytes was measured by chemiluminescence method. *Results*. At the highest used concentration of iron ions Fe^3+^ (100 *μ*M), free radicals production by peritoneal phagocytes was reduced by 90% compared to control. *Conclusions*. Iron overload may increase the risk of infectious complications in ESRD patients.

## 1. Introduction

Anemia is a common complication of end-stage renal disease (ESRD) and results from decreased erythropoietin (EPO) production, reduced bone marrow response to EPO, shortened red blood cell survival, and chronic iron loss. Anemia can be treated by administration of erythropoiesis-stimulating agents, that is, EPO [1].

The efficacy of EPO treatment in dialysis patients is determined mainly by the availability of iron [2]. Hence, intravenous iron supplementation is recommended in ESRD patients to support erythropoiesis [3]. However, iron supplementation can cause iron overload, occurring typically when the plasma iron content exceeds the iron-binding capacity of transferrin. Iron overload can also be a consequence of increased erythrocyte destruction [4].

Iron and its binding proteins have immunoregulatory properties, and altering the balances in immune system by iron overload or deficiency may produce detrimental physiological effects [5]. As infection contributes greatly to mortality and morbidity in ESRD patients [6], the potential for iron therapy to impair immune system and thus to increase the risk of infection deserves special consideration.

Reactive oxygen species produced by phagocytic leukocytes (macrophages, monocytes, and neutrophils) play a primary role in antimicrobial defense of the host. Phagocytosis of bacteria, viruses, and so forth is accompanied by a sharp increase in oxygen uptake, a process known as “respiratory burst,” resulting in the production of the toxic oxygen compounds, that is, H_2_O_2_, hydroxyl radical, and superoxide anion, which kill phagocyted microorganisms [7].

The aim of the study was to investigate *in vitro *the effects of iron ions Fe^3+^ on the respiratory burst of phagocytes isolated from peritoneal effluents of continuous ambulatory peritoneal dialysis (CAPD) patients.

## 2. Methods

### 2.1. Isolation of Peritoneal Phagocytes

Peritoneal phagocytes were obtained from CAPD patients not receiving iron and/or EPO cotreatment and not suffering from any infection during the study. Cells were isolated from peritoneal overnight dwell time effluents by centrifugation (1200 ×g, 10 min, 4°C), followed by removal of the contaminating erythrocytes by hypotonic lysis. To harvest sufficient number of cells, effluents of five different patients were used for obtainment of each cell pool. Following isolation, cells were resuspended in Krebs-Ringer buffer, counted, and analysed using May-Grünwald Giemsa staining performed concomitantly with the trypan blue exclusion viability test.

The study was approved by the ethics committee of Jagiellonian University in Cracow, and informed consent was obtained from each of the patients.

### 2.2. Measurements of Respiratory Burst

Respiratory burst of peritoneal phagocytes was measured using chemiluminescence (CL) method. Briefly, cells were plated in 96-well white plates (1.5 × 10^5^/well) and incubated (30 min, 37°C) in the presence or absence of urea (5–50 mM), creatinine (50–1000 *μ*M), or iron ions Fe^3+^ (10–100 *μ*M; as FeCl_3_ solution), before addition of Krebs-Ringer buffer containing luminol (5-amino-2,3-dihydro-1,4-phthalazinedione; 2.3 mM) and glucose (5 mM). In the separate series of experiments, cells were exposed for 20 min to lipopolysaccharide (LPS; *E. coli* 0111:B4; 100 ng/mL) prior to the treatment with the above-mentioned agents. Respiratory burst was initiated by addition of latex beads suspension (polystyrene, 0.8 *μ*m, 3% aqueous suspension; 10 *μ*L/well). CL was measured for 60 min at 37°C using Microlumat LB96P luminometer (Berthold, Austria).

### 2.3. Statistics

All measurements were performed in triplicates. Respiratory burst results are expressed as area under curve (AUC), being the difference between time-response curves of CL intensities recorded in the presence and absence of latex. Results are given as mean ± SEM, calculated on the basis of five independent experiments, each experiment being performed on cells pooled from dialysates of five patients. Unless indicated otherwise, statistical analyses were performed on nonnormalized data using one-way ANOVA followed by Tukey-Kramer multiple comparison test. IC_25_ and IC_50_ values were estimated based on interpolations of data from concentration-response curves. *, **, and *** indicate *P* < 0.05, 0.01 and 0.001, respectively.

## 3. Results

In the present study, the effects of iron ions Fe^3+^ on latex-induced respiratory burst of peritoneal phagocytes were investigated. As bacterial infection is considered as a major complication of CAPD [8], respiratory burst was also measured in the presence of LPS, one of the most powerful bacterial virulence factors with proinflammatory properties [9]. The effects of iron were compared with phagocyte response to two classical uremic toxins, that is, urea and creatinine.

Peritoneal cell populations used in the study contained 42–52% macrophages, 36–42% lymphocytes, and 5–7% neutrophils, and their overall viability was 93–97%. The highest respiratory burst intensities were observed for 1.5 × 10^5^ cells/well and 10 *μ*L latex suspension/well (data not shown), and these conditions were used in subsequent experiments.

**Figure 1:**
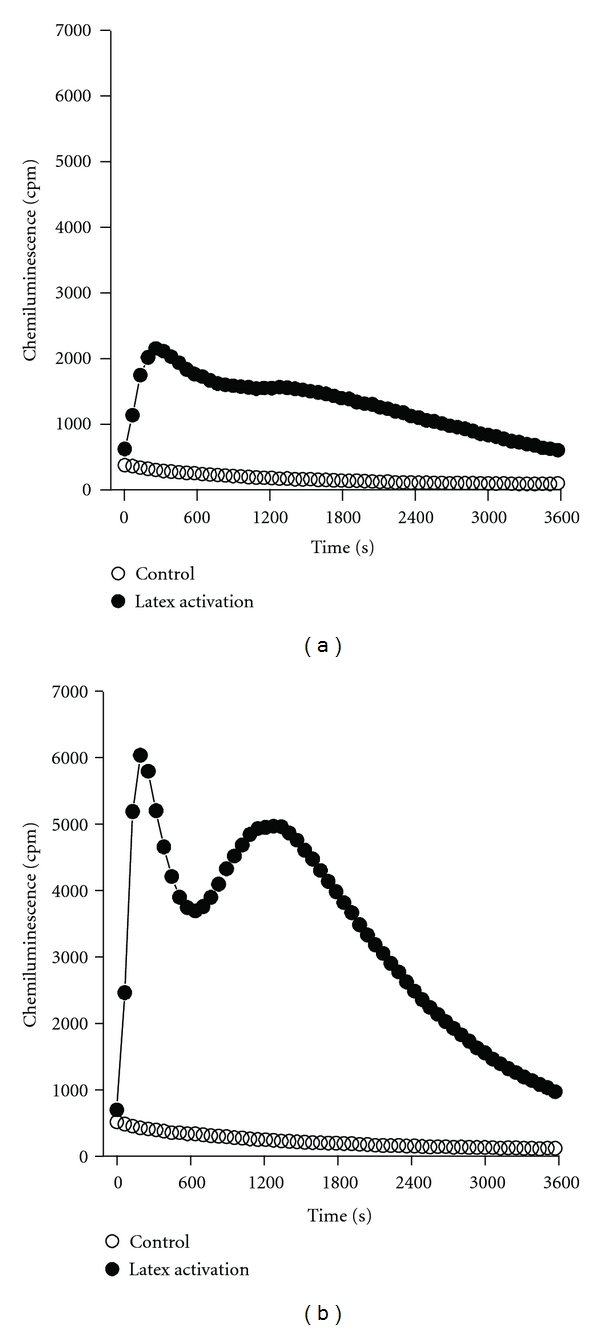
Respiratory burst of nonprimed (a) and LPS-primed (100 ng/mL, 20 min) (b) peritoneal phagocytes activated by latex beads suspension (10 *μ*L). Representative time-response curves of CL intensities recorded for 60 min at 37°C in the presence (●) and absence (○) of latex are shown.

LPS was found to be a strong primer of latex-induced respiratory burst. Figures 1(a) and 1(b) show time-response curves of CL for nonprimed and LPS-primed (100 ng/mL, 20 min) cells, respectively, with the AUC value calculated for the primed cells being ~2.5 × higher than in the absence of LPS.

**Figure 2:**
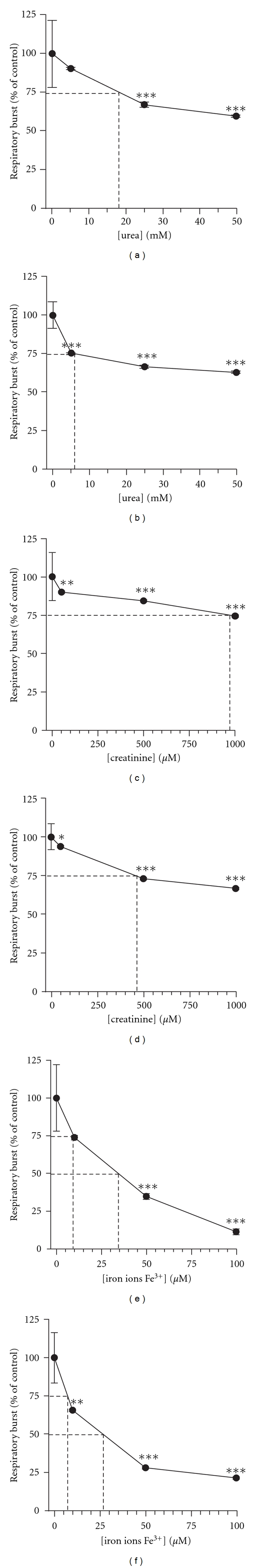
Effects of urea ((a) and (b)), creatinine ((c) and (d)), and iron ions Fe^3+^ ((e) and (f)) on respiratory burst of peritoneal phagocytes. Dose-response curves showing the intensities of respiratory burst calculated from AUC values for nonprimed ((a), (c), and (e)) and LPS-primed (100 ng/mL, 20 min; (b), (d), and (f)) cells. Cells were exposed to tested agents for 30 min prior to CL measurements. Results are given as mean ± SEM relative to controls on the basis of five independent experiments.

Iron ions Fe^3+^, urea, and creatinine were used at the concentrations ranging from physiological to maximal (but still observed in uremic patients). All these agents inhibited respiratory burst of both nonprimed (Figures 2(a), 2(c), and 2(e)) and LPS-primed (Figures 2(b), 2(d), and 2(f)) cells in a concentration-dependent manner. However, the sensitivities of the cells to tested agents were highly variable. Thus, for nonprimed cells, the highest used concentrations of agents caused an average reduction of respiratory burst to ~59, 74, and 11% relative to controls, for urea, creatinine, and iron ions Fe^3+^, respectively. Likewise, at the highest concentrations used, respiratory burst of LPS-primed cells was most strongly reduced by iron ions Fe^3+^ (~21% of control), whereas both urea and creatinine caused milder inhibitions (~63 and 66% of control, resp.).  Table 1 presents IC_25_ and IC_50_ values for the estimated effects of tested agents on respiratory burst. Only iron ions Fe^3+^ exhibited the IC_50_ values below the highest used concentration (100 *μ*M), for both nonprimed and LPS-primed cells whereas all tested agents exhibited IC_25_ values within employed concentration ranges.

## 4. Discussion

Achieving and maintaining iron sufficiency is crucial for the efficacy of EPO treatment in dialysis patients [2]. Available reports on the effects of iron supplementation on immunity of ESRD patients are inconclusive. Several large-scale prospective studies showed no relationship between risk of bacteremia and iron administration [10, 11]. In contrast, retrospective trial in hemodialysis patients reported a possible increase in infection rate associated with iron therapy [12]. As the biologically active iron plays an important role in the immunity [5], the controversy surrounding the effects of iron supplementation on risk of infections in ESRD patients requires special attention.

In this study, we investigated *in vitro *effects of iron on respiratory burst of phagocytes obtained from CAPD patients. Investigations were performed on both nonprimed and LPS-primed phagocytes and compared to the effects of urea and creatinine. The lowest concentrations of urea and creatinine used in our study reflected the physiological plasma levels found in healthy subjects while middle and the highest concentrations were within the range observed in ESRD patients [13]. Iron was used at the concentrations comparable with the plasma levels of non-transferrin-bound Fe^3+^ in CAPD patients (10 mM) or in iron overload patients (50–100 mM) [14].

Both urea and creatinine inhibited the respiratory burst of phagocytes in a concentration-dependent manner, even at concentrations comparable to physiological. This observation is in agreement with the results obtained by Daniels et al. showing the inhibitory effects of low-molecular-weight constituents of peritoneal dialysis effluent on respiratory burst of PMNL *in vitro *[15]. The effects of non-transferrin-bound Fe^3+^ on respiratory burst were much more pronounced as compared to urea and creatinine. At the highest used concentration of iron, free radicals production by peritoneal phagocytes was reduced by 90%.

The mechanism by which iron inhibits respiratory burst of peritoneal phagocytes remains unclear. A possible explanation could be peroxidation of membrane lipids mediated by iron ions Fe^3+^ as some products of lipid peroxidation have been reported to suppress respiratory burst of macrophages [16]. Further investigations are needed to verify this hypothesis. Future studies should also address the possibility of additive or synergistic interactions between uremic toxins and iron ions Fe^3+^.

The results obtained in this study suggest that iron supplementation can suppress respiratory burst of phagocytes. As the iron deficiency can also impair immune system, careful assessing of iron status in ESRD patients can be of importance to reduce the risk of infections. However, data obtained *in vitro* must be considered with appropriate limitations when we try to extrapolate them directly to *in vivo *situation.

## Figures and Tables

**Table 1:** IC_25_ and IC_50_ values for the effects of urea, creatinine, and iron on respiratory burst of nonprimed and LPS-primed peritoneal phagocytes.

Tested agent	Used phagocytes	IC_25_		IC_50_	
Urea	Nonprimed	18.3 mM	(17.3–19.5)	>50 mM	—
	LPS-Primed	6.1 mM	(5.3–6.9)	>50 mM	—
Creatinine	Nonprimed	973.3 *μ*M	(940.1–1007.6)	>1000 *μ*M	—
	LPS-Primed	455.3 *μ*M	(444.9–465.8)	>1000 *μ*M	—
Iron ions Fe^3+^	Nonprimed	9.5 mM	(5.3–10.0)	34.5 mM	(32.8–36.2)
	LPS-Primed	7.3 mM	(4.7–8.3)	26.8 mM	(26.2–27.5)

Numbers in parentheses indicate interpolated values for IC_25_ or IC_50_ ± SEM (based on AUC values from five independent experiments).

## References

[1] Valderrâbano F (1996). Erythropoietin in chronic renal failure. *Kidney International*.

[2] Macdougall IC, Chandler G, Elston O, Harchowal J (1999). Beneficial effects of adopting an aggressive intravenous iron policy in a hemodialysis unit. *American Journal of Kidney Diseases*.

[3] Eschbach J, DeOreo P, Adamson J (1997). NKF-DOQI clinical practice guidelines for the treatment of anemia of chronic renal failure. *American Journal of Kidney Diseases*.

[4] Anderson GJ (2007). Mechanisms of iron loading and toxicity. *American Journal of Hematology*.

[5] Walker EM, Walker SM (2000). Eeffects of iron overload on the immune system. *Annals of Clinical and Laboratory Science*.

[6] Powe NR, Jaar B, Furth SL, Hermann J, Briggs W (1999). Septicemia in dialysis patients: incidence, risk factors, and prognosis. *Kidney International*.

[7] Forman HJ, Torres M (2001). Signaling by the respiratory burst in macrophages. *IUBMB Life*.

[8] Davenport A (2009). Peritonitis remains the major clinical complication of peritoneal dialysis: the London, UK, peritonitis audit 2002-2003. *Peritoneal Dialysis International*.

[9] Chow JC, Young DW, Golenbock DT, Christ WJ, Gusovsky F (1999). Toll-like receptor-4 mediates lipopolysaccharide-induced signal transduction. *Journal of Biological Chemistry*.

[10] Aronoff GR, Bennett WM, Blumenthal S (2004). Iron sucrose in hemodialysis patients: safety of replacement and maintenance regimens. *Kidney International*.

[11] Furuland H, Linde T, Ahlmén J, Christensson A, Strömbom U, Danielson BG (2003). A randomized controlled trial of haemoglobin normalization with epoetin alfa in pre-dialysis and dialysis patients. *Nephrology Dialysis Transplantation*.

[12] Canziani ME, Yumiya ST, Rangel EB, Manfredi SR, Neto MC, Draibe SA (2001). Risk of bacterial infection in patients under intravenous iron therapy: dose versus length of treatment. *Artificial Organs*.

[13] May RC, Mitch WE, Brenner BM, Rector FC (1996). Pathophysiology of uremia. *The Kidney*.

[14] Kooistra MP, Marx JJM (1998). The absorption of iron is disturbed in recombinant human erythropoietin-treated peritoneal dialysis patients. *Nephrology Dialysis Transplantation*.

[15] Daniels I, Bhatia KS, Porter CJ (1996). Hydrogen peroxide generation by polymorphonuclear leukocytes exposed to peritoneal dialysis effluent. *Clinical and Diagnostic Laboratory Immunology*.

[16] Wilhelm J, Skoumalová A, Vytášek R, Fišárková B, Hitka P, Vajner L (2005). Erythrocyte membranes inhibit respiratory burst and protein nitration during phagocytosis by macrophages. *Physiological Research*.

